# Transcranial sonography with clinical and demographic characteristics to predict cognitive impairment in PD: a longitudinal study

**DOI:** 10.1186/s12883-023-03057-1

**Published:** 2023-01-13

**Authors:** Zhiguang Chen, Wei Zhang, Wen He, Yang Guang, Tengfei Yu, Yue Du, Rui Li

**Affiliations:** Department of Ultrasound, Beijing Tiantan Hospital, Capital Medical University, Beijing, 100050 China

**Keywords:** Parkinson’s disease, Cognitive impairment, Risk factors, Logistic regression analysis

## Abstract

**Background:**

Parkinson’s disease (PD) is a neurodegenerative disease and is clinically characterized by a series of motor symptoms (MS) and nonmotor symptoms (NMS). NMS often appear before MS, while cognitive impairment mostly occurs within a few years after the diagnosis of PD. Therefore, we aimed to predict the risk factors for cognitive impairment (CI) in PD patients based on transcranial sonography, clinical symptoms, and demographic characteristics.

**Methods:**

Based on the occurrence time of CI, a total of 172 PD patients were divided into non-CI (N-CI, *n* = 48), CI at the first treatment (F-CI, *n* = 58), and CI at the last treatment (L-CI, *n* = 66) groups. Clinical data (including MS and NMS) and ultrasonic data of all patients at the first treatment and the last treatment were collected retrospectively. Independent samples *t* tests were used to compare continuous data, and chi-square tests were used to compare categorical data. The risk factors for CI and Parkinson’s disease dementia were identified by logistic regression analysis, and an ROC curve was established to explore the diagnostic efficacy.

**Results:**

1) The age of onset, first treatment and smoking history of CI patients were significantly different from those of N-CI patients. When age of first treatment ≥61 years was considered the boundary value to diagnose CI, the sensitivity and specificity were 77.40 and 66.70%, respectively. 2) The severity of depression was significantly different between F-CI and N-CI patients at the first treatment, while the cumulative and new or aggravated memory deficit was significantly different between the L-CI and N-CI patients at the last treatment. 3) There was a significant difference in TCS grading between the first and last treatment in L-CI patients. 4) Depression, sexual dysfunction, and olfactory dysfunction in NMS were independent risk factors for CI during the last treatment. 5) The sensitivity and specificity of predicting CI in PD patients were 81.80 and 64.60%, respectively.

**Conclusions:**

PD patients with CI were older, and most of them had a history of smoking. Furthermore, there was good diagnostic efficiency for predicting CI in PD via TCS combined with clinical characteristics (especially NMS).

## Introduction

Parkinson’s disease (PD) is a progressive neurodegenerative disorder that is characterized by bradykinesia, resting tremors, and rigidity. Motor symptoms (MS) are the core feature of PD diagnosis, but nonmotor symptoms (NMS) often appear before MS and are considered a new diagnostic classification called prodromal PD [1]. NMS include mental symptoms, autonomic symptoms, and sensory abnormalities. NMS are not only related to the early diagnosis of PD but also closely related to prognosis [2]. For example, a neurophysiological review of rapid eye movement (REM) sleep behaviour disorder (RBD) suggests that RBD may lead to cognitive impairment (CI) in PD [3].

Dementia is mostly associated with diseases of the central nervous system (CNS) [4]. In PD patients with a history of over 10 years, the incidence rate of dementia exceeds 75%. Mild cognitive impairment (MCI) refers to a transitional stage in PD patients who have cognitive impairment but do not develop PD dementia (PDD). It is also an important factor in the disability rate in PD [5]. Janvin et al. [6] have shown that almost all patients with MCI will eventually meet the diagnostic criteria of PDD. Therefore, early identification of potential risk factors for MCI and prediction of PDD in PD patients are crucial for clinical diagnosis and treatment.

Substance nigra hyperechogenicity (SNH) in transcranial sonography (TCS) is common in most PD patients and has high sensitivity and specificity. However, SNH can still occur in normal subjects or in other CNS-related diseases, such as Gaucher’s disease [7]. At present, the mechanism of hyperechogenicity is still unclear. Some studies have shown that the formation of hyperechogenicity may be related to the deposition of ferritin [7–9], which has been verified by quantitative susceptibility mapping [10]. Additionally, the hyperechogenicity range is positively correlated with the extinction degree of SN dopaminergic neurons, and the activation of microglia is related to the formation of hyperechogenicity [11]. Compared with other imaging examinations, TCS has the advantages of simplicity and rapidity, especially in the emergency department. Godani and his colleagues [12] reported that TCS was used to examine an emergency patient with unusual gait disorder, which quickly provided the diagnosis results of neurodegenerative motor disorder, thereby avoiding the delay of the patient’s condition.

MS and NMS, especially NMS, have been the focus of research on the prognosis of PD patients in recent years, and whether these symptoms are related to changes in the substantia nigra is our concern. As a real-time, simple, economic, nonradioactive examination method, TCS plays an important role in the diagnosis and follow-up of PD patients [13]. To the best of our knowledge, there are few reports on the prediction of PD using TCS combined with clinical data, and the application is limited [14–17]. Therefore, we aimed to use the results of at least two TCS imaging studies, changes in clinical symptoms (MS and NMS), and demographic data to evaluate and predict the possibility and risk factors for CI and dementia in PD patients.

## Materials and methods

### Ethics declarations

#### Ethics approval and consent to participate

The Ethics Committee of the Beijing Tiantan Hospital Affiliated to Capital Medical University (Beijing, China) approved the study protocol. The study was carried out according to the tenets of the Declaration of Helsinki. All methods were carried out in accordance with relevant guidelines and regulations.

### Data collection and patient information

Data were collected from all inpatients admitted to the Ward of Dyskinesia and Cognitive Disorders, Beijing Tiantan Hospital, Capital Medical University, from April 2019 to April 2021. In all, 988 of the 1540 inpatients were excluded because of Parkinson’s syndrome, essential tremor (ET), progressive supranuclear palsy (PSP), multiple system atrophy (MSA), vascular PD, and Alzheimer’s disease. The remaining 552 patients were clinically diagnosed with PD. Of these patients, 335 had only one hospitalization experience. Of the remaining 217 patients, 18, 15, and 12 patients who had incomplete TCS results, incomplete clinical history, and a < 6-month interval between two hospitalizations, respectively, were excluded. Finally, 172 eligible patients with PD were included in this study.

The inclusion criteria were as follows: (i) the clinical diagnosis was PD; (ii) the MS and NMS records were complete; (iii) TCS examination was completed during both hospitalizations, and the SN could be clearly displayed and graded (during hospitalization, the time between TCS and hospitalization was no more than 2 weeks); and (iv) the interval time between two hospitalizations was ≥6 months.

The exclusion criteria were as follows: (i) the clinical diagnosis was non-PD; (ii) the SN of TCS examination was not clear; and (iii) the clinical history was incomplete.

**Fig. 1:**
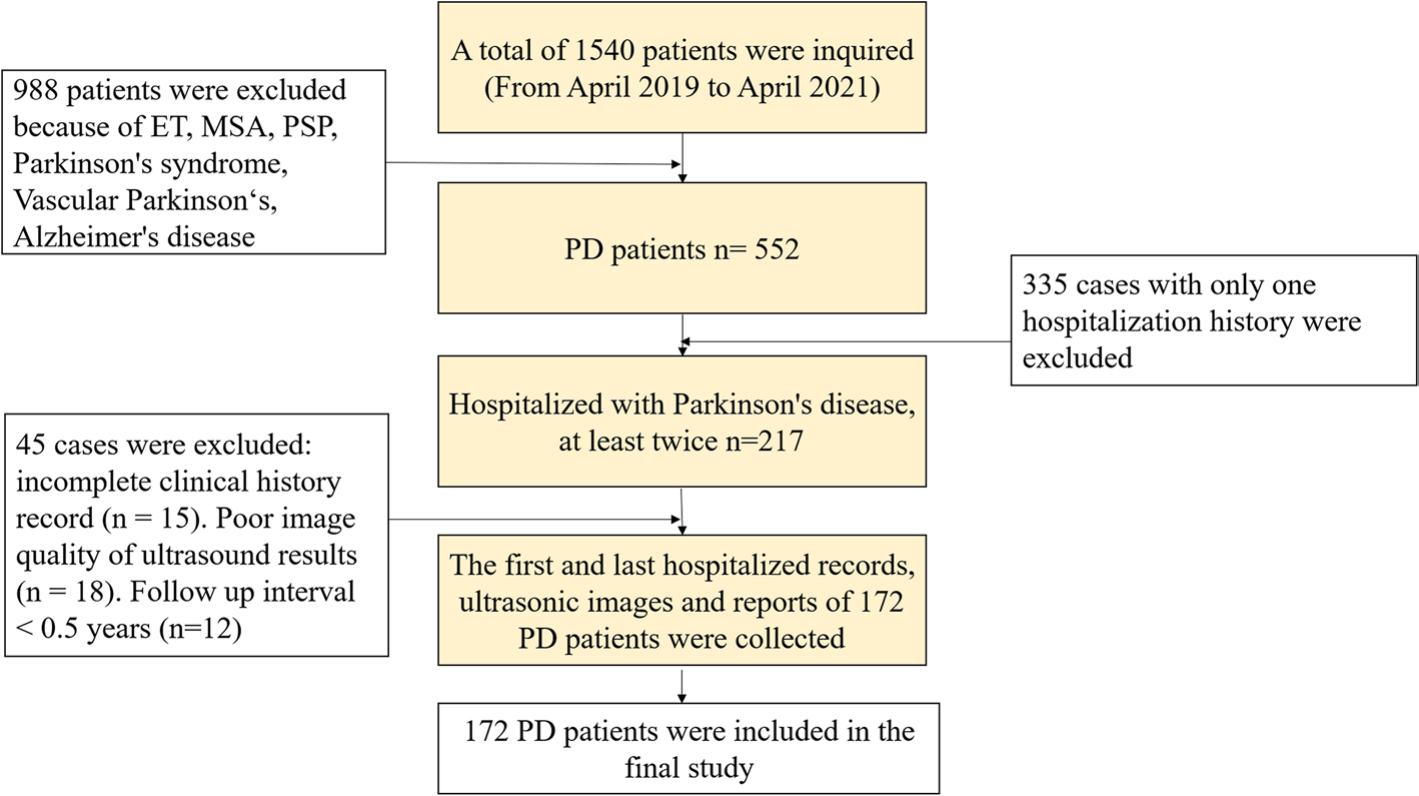
Patient flowchart. Exclusion criteria and inclusion process. PD, Parkinson’s Disease. ET, essential tremor. PSP, progressive supranuclear palsy. MSA, multiple system atrophy

The process of patient selection is shown in Fig. 1.

### Diagnosis of PD

All patients with PD included in this study had clinically established PD, and the diagnostic criteria were as follows: 1) there were no absolute exclusion criteria; 2) at least 2 supporting standards existed; and 3) there were no red flags [18].

### Clinical data collection

All subjects underwent a thorough neurological examination including the motor part of the Unified PD Rating Scale (UPDRS-III). The Mini Mental State Examination (MMSE) and Montreal Cognitive Assessment (MoCA) were used to screen for CI. The Pittsburgh Sleep Quality Index (PSQI) was used to evaluate sleep disorders, the Hamilton Anxiety Scale (HAMA) was used to evaluate anxiety, the Hamilton Depression Scale (HAMD) was used to evaluate depression, and the Drooling Severity and Frequency Scale (DSFS) was used to evaluate salivation. Postural hypotension was evaluated by blood pressure measurement in the supine position 1 min, 3 min, and 5 min after standing up. According to a patient’s history and > 3 failures on the 12-item smell identification test from Sniffin’ Sticks, olfactory dysfunction was evaluated. The Brief Pain Inventory (BPI) was used to evaluate pain. The Restless Legs Syndrome Severity Rating Scale (RLSRS) was used to evaluate restless legs syndrome (RLS).

### Transcranial sonography

The patient was placed in the supine position with full exposure of the temporal window. A Philips (Canon Aplio i900) S5-1 MHz phased array probe was used for scanning, and the depth and brightness of the instrument were adjusted according to the patient’s condition. Two sonographers with at least 5 years of experience in the diagnosis of SN lesions performed the ultrasound examination, and the hyperechoic areas of the SN were depicted and measured in a double-blind manner without knowing the patient’s clinical diagnosis. The echo of the SN was graded according to Bartova et al. [19].

### Functional MRI (fMRI)

All subjects were scanned on a 3.0 T MRI scanner (GE Health, USA) equipped with an eight-channel head coil to obtain MRI images, including T1-weighted, T2-weighted, and diffusion-weighted images. During MRI scanning, the head was fixed, and earplugs were used to reduce the impact of angiography on patients. When the image quality was poor, the scanning was repeated. Regions of interest (ROIs) were traced, and an analysis was carried out to calculate tensor fractional anisotropy (FA), axial diffusivity (AD), radial diffusivity (RD), and mean diffusivity (MD).

### Data analysis

Statistical analyses were performed using SPSS software (version 25.0, (IBM Corporation, Armonk, NY, USA). We analysed the demographic and basic clinical information of 172 PD patients. Continuous data are expressed as the mean ± standard deviation (SD). Independent samples *t* tests were used to compare the age of onset, age of first treatment, and duration of disease. Sex and ethnicity family history, smoking history, drinking history, hypertension, hyperlipidaemia, diabetes, and history of head injury and carbon monoxide poisoning were analysed by chi-square test. Furthermore, we used the chi-square test to analyse the clinical symptoms of PD patients with and without CI at the first treatment and the accumulated and new or aggravated clinical symptoms at the last treatment.

Cohen’s kappa consistency test was used to evaluate the judgement and grading of hyperechoic results in the SN by two sonographers. Parameters with *P* < 0.1 were included in the logistic regression analysis, and the ROC curve was established to analyse the diagnostic efficiency. Finally, odds ratios (ORs) were calculated for the clinical symptoms and ultrasonic signs of dementia and nondementia in patients with CI.

## Results

### Clinical demographic results

The 172 PD patients were divided into three groups based on the time of appearance of CI: CI occurring at the first treatment (F-CI, *n* = 58); CI occurring at the last treatment (L-CI, *n* = 66); and no CI during the entire duration of follow-up (N-CI, *n* = 48). In this study, the age of onset was equal to the age of first treatment minus the duration of disease. There was a significant difference in the age of onset, age of first treatment, and smoking history between the N-CI and CI groups (F-CI and L-CI), but no difference was noted between the F-CI and L-CI groups (Table 1).

**Table 1:** Demographic and clinical characteristics

	N-CI (*n* = 48)	F-CI (*n* = 58)	L-CI (*n* = 66)	*P*
Age of onset (years) Mean ± SD	14–77 (53.20 ± 11.29)	47–73 (60.08 ± 4.78)	32.5–75 (59.82 ± 10.28)	**<0.001**^**a**^ 0.862^b^
Age of first treatment (years) Mean ± SD	29–78 (58.00 ± 10.58)	51–78 (64.53 ± 4.36)	33–80 (64.89 ± 10.92)	**<0.001**^**a**^ 0.816^b^
Gender				
Male	34	35	46	0.429^c^
Female	14	23	20	
Ethnicity				
Han	46	57	64	0.749^e^
Others	2	1	2	
Family history	2	4	3	0.787^e^
Smoking history	9	15	29	**0.010**^**c**^**/0.005**^**cd**^
Drinking history	10	4	9	0.105^e^
Hypertension	17	20	27	0.727^c^
Diabetes	9	15	18	0.551^c^
Hyperlipidemia	10	11	14	0.948^c^
History of head injury	3	5	6	0.849^e^
History of carbon monoxide poisoning	6	5	11	0.407^c^
Duration of disease (years) Mean ± SD	0.5-20 (4.80 ± 4.38)	0.5-20 (4.46 ± 4.14)	0.5-30 (5.08 ± 5.19)	0.763^a^

^a^Single factor ANQVA analysis

^b^Independent sample t test, comparison of F-CI and L-CI

^c^Chi square test

^d^Comparison of N-CI and L-CI

^e^Continuity correction of chi square test

**Fig. 2:**
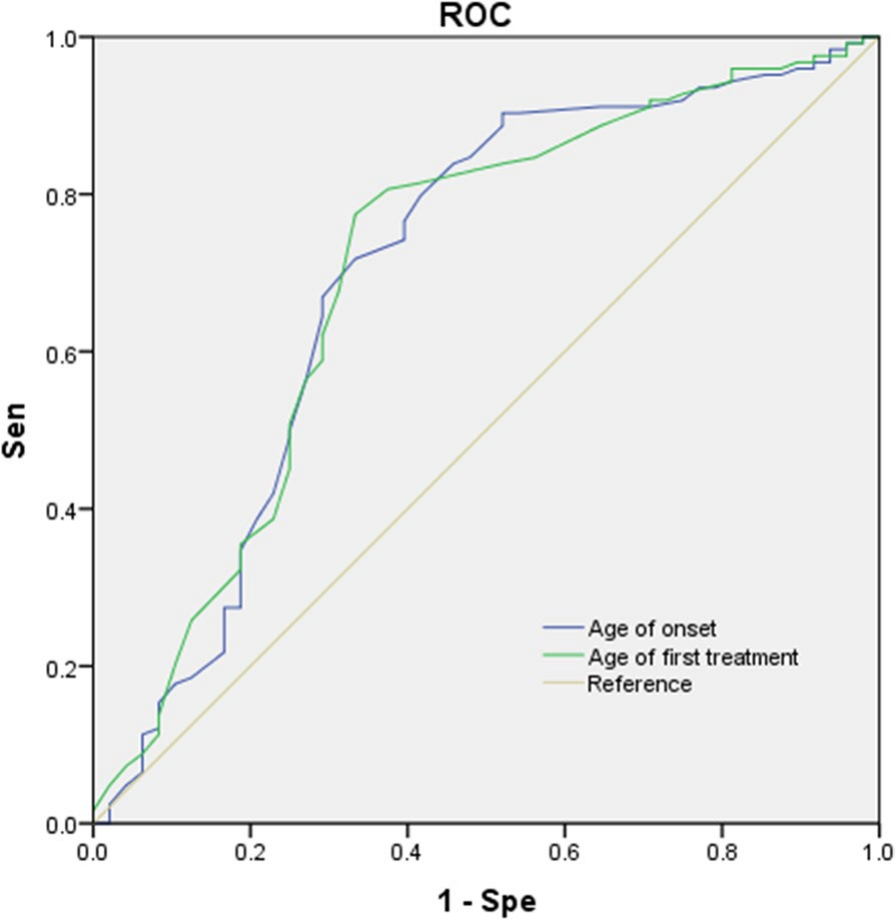
ROC curve of N-CI and CI (F-CI and L-CI). The area under ROC curve (AUC) of age of onset and age of first treatment were 0.702 (95%CI: 0.606–0.798) and 0.704 (95%CI: 0.610–0.798), respectively

We used single-factor ANOVA to compare the age of onset, age of first treatment, and duration of disease. The area under the ROC curve (AUC) for age at first treatment was the largest (Fig. 2). When the age of first treatment ≥61 years was considered the threshold value for PD diagnosis in patients with CI, the sensitivity and specificity were 77.40 and 66.70%, respectively.

### Comparison of MS and NMS among subgroups

#### Comparison of F-CI and N-CI and non-FCI (L-CI and N-CI) clinical symptoms at the first treatment

We carried out detailed statistical analysis on the clinical symptoms of PD patients at the first treatment and compared the MS and NMS of F-CI and N-CI, F-CI and non-FCI (which refers to PD patients without CI at the first treatment, including L-CI and N-CI). As shown in Table 2, there was a significant difference in depression between F-CI and N-CI, which shows that depression is more common in patients with CI at the first treatment than in those without CI during treatments.

**Table 2:** Comparison of clinical symptoms of patients with F-CI, N-CI and N-FCI at the first treatment

Clinical symptoms	F-CI (*n* = 58)	N-FCI (*n* = 114)	N-CI (*n* = 48)	P1	P2
MS					
Bradykinesia	58	114	48	NA	NA
Rest tremor	49	98	41	0.794^c^	0.894^c^
Rigidity	36	66	31	0.598^c^	0.789^c^
Mental symptoms					
Memory deficit	31	48	18	0.158^c^	0.101^c^
RCP	5	12	3	0.692^c^	0.928^e^
Cognitive impairment	58	0	0	NA	NA
Somnipathy	14	17	7	0.137^c^	0.219^c^
Anxiety	25	41	13	0.363^c^	0.087^c^
Depression	21	36	9	0.542^c^	**0.047**^c^
Apathy	5	6	2	0.395^c^	0.599^e^
Autonomic symptoms					
Constipation	33	71	26	0.495^c^	0.778^c^
Hyperhidrosis	2	1	1	0.547^e^	NA*
Postural hypotension	10	19	6	0.924^c^	0.497^c^
Dysuria	31	57	23	0.669^c^	0.571^c^
Salivation	11	15	4	0.315^c^	0.199^e^
Sexual dysfunction	5	13	9	0.573^c^	0.125^c^
Sensory disturbance					
Olfactory dysfunction	18	36	20	0.942^c^	0.256^c^
Pain	11	19	8	0.707^c^	0.759^c^
Spasm	0	1	1	NA	NA
Numbness	1	5	2	0.646^e^	0.868^c^
RLS	2	4	1	NA*	NA*

P1: Comparison of MS and NMS between F-CI and N-FCI at the first treatment

P2: Comparison of MS and NMS between F-CI and N-CI at the first treatment

*RLS* Restless legs syndrome, *MS* Motor symptoms, *RCP* Reduced computing power, *NA* Not available

NA* Not available after continuity correction, *P* = 1

^c^Chi square test

^e^Continuity correction of chi square test

Upon comparing the F-CI with the N-CI, the *P* values of depression and anxiety were 0.047 and 0.087, respectively, which were included in the logistic regression analysis.

#### Comparison of clinical symptoms at the first treatment and cumulative and newly added symptoms at the last treatment between L-CI and N-CI

We compared the clinical symptoms of L-CI and N-CI at the first treatment. There was a significant difference in depression and olfactory dysfunction between the L-CI and N-CI groups (Table 3). Accordingly, we analysed the new or aggravated symptoms of L-CI and N-CI patients at the last treatment and compared the differences in the total clinical symptoms. The results suggest that there is a significant difference in total symptoms between L-CI and N-CI in memory deficit and reduced computing power (RCP). New or aggravated memory deficits were more common in L-CI patients than in N-CI patients (*P* = 0.016).

**Table 3:** Comparison of total, baseline and new or aggravated clinical symptoms between L-CI and N-CI

Clinical symptoms	L-CI *(*n = 66)	N-CI (*n* = 48)	P1	L-CI t/n (*n* = 66)	N-CI t/n (*n* = 48)	P2	P3
MS							
Bradykinesia	66	48	NA	66/11	48/10	NA	0.571^c^
Rest tremor	57	41	0.886^c^	58/7	43/9	0.777^c^	0.123^c^
Rigidity	35	31	0.217^c^	37/8	34/10	0.108^c^	0.208^c^
Mental symptoms							
Memory deficit	30	18	0.396^c^	55/28	26/10	**0.001**^**c**^	**0.016**^**c**^
RCP	9	3	0.337^e^	22/13	7/4	**0.023**^**c**^	0.157^c^
Cognitive impairment	0	0	NA	66/0	0/0	NA	NA
Somnipathy	10	7	0.933^c^	23/13	16/9	0.866^c^	0.899^c^
Anxiety	28	13	0.092^c^	33/7	22/9	0.660^c^	0.123^c^
Depression	27	9	**0.012**^**c**^	35/10	17/9	0.062^c^	0.611^c^
Apathy	4	2	0.982^e^	5/1	2/0	0.724^e^	NA
Autonomic symptoms							
Constipation	45	26	0.127^c^	54/15	35/9	0.257^c^	0.607^c^
Hyperhidrosis	0	1	NA	1/1	2/1	0.779^e^	NA*
Postural hypotension	13	6	0.309^c^	25/13	17/11	0.788^c^	0.677^c^
Dysuria	34	23	0.704^c^	44/11	34/11	0.637^c^	0.404^c^
Salivation	11	4	0.308^e^	16/5	8/4	0.327^c^	NA*
Sexual dysfunction	4	9	0.071^e^	6/2	10/1	0.075^c^	NA
Sensory disturbance							
Olfactory dysfunction	16	20	**0.048**^**c**^	27/11	26/6	0.161^c^	0.537^c^
Pain	11	8	NA*	16/6	11/5	0.896^c^	0.813^c^
Spasm	0	1	NA	0/0	1/0	NA	NA
Numbness	3	2	NA*	4/2	2/0	0.982^e^	NA
RLS	3	1	0.849^e^	3/0	1/0	0.873^e^	NA

P1: Comparison of MS and NMS between L-CI and N-CI at the first treatment

P2: Comparison of total clinical symptoms at the last treatment between L-CI and N-CI

P3: Comparison of newly increased or aggravated clinical symptoms between L-CI and N-CI at the last treatment

*t/n* total/ new or aggravated

^c^Chi square test

^e^Continuity correction of chi square test

Statistically significant clinical symptoms (depression, olfactory dysfunction, memory deficit, RCP, all *P* < 0.05) and sexual dysfunction and anxiety (0.05 ≤ *P* < 0.1) were included in the logistic regression analysis.

### Comparison of TCS results between the first and last examinations

**Fig. 3:**
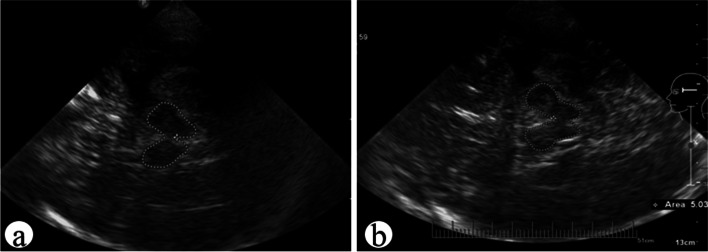
Male, 65 years old. **a** Midbrain area was 4.99cm^2^ and SN grading 2 at the first treatment; **b** After 3 years, the symptoms of rigidity and resting tremor worsened, and there is a decrease in calculation and memory. When hospitalized again, the midbrain area was 5.03cm^2^ and the SN grading was 3; At the last discharge diagnosis, the patient had cognitive impairment

TCS was performed in PD patients during the first and last hospitalizations. In almost all patients, the midbrain area, hyperechoic area, and echo grading of the SN were recorded. Compared with the first TCS, the increased grade of SN in PD patients was significantly greater than the decreased grade (*P* = 0.001) (Fig. 3, Table 4).

**Table 4:** Comparison of first and Last TCS results

		N-CI(n = 48)	F-CI (n = 58)	L-CI (n = 66)	P1	P2	P3
Interval time (Years) Mean ± SD		0.50-7.67 (3.09 ± 1.76)	0.50-8.33 (3.34 ± 1.86)	1.00-8.00 (2.77 ± 1.84)	0.232^a^	NA	NA
Midbrain area							
Total	First	4.833 ± 0.667			0.01^a^	NA	NA
	Last	4.999 ± 0.777					
Subgroup	First	4.854 ± 0.708	4.782 ± 0.642	4.863 ± 0.665	NA	NA	0.141^a^ /**0.018**^**a**^ /0.434^a^
	Last	4.977 ± 0.733	5.071 ± 0.725	4.952 ± 0.856			
Ultrasound grading							
First	SNH+	22	20	29	0.07^c^	NA	0.307^c^ /0.186^c^**/0.023**^c^
Last	SNH+	27	27	42			
Ultrasound grading changes					**0.001**^**c**^		
	Up	9	13	19	NA	0.899^c^/0.219^c^/0.418^c^	NA
	Down	4	8	6	NA	0.565^e^/NA^e^/0.409^c^	NA

P1: Overall comparison among the three groups

P2: The comparison between groups, from left to right was N-CI & F-CI, N-CI & L-CI and F-CI & L-CI

P3: The difference between the first and last ultrasonic signs in each group, from left to right was N-CI, F-CI and L-CI

Interval time, the interval between two ultrasound examinations

^a^Single factor ANQVA analysis

^c^Chi square test

There was no statistically significant difference in the ultrasonic examination time interval among the three groups of PD patients. Interestingly, there was a difference in the midbrain area between the last and first examinations in patients with F-CI but not in patients with L-CI and N-LC. The grading of the last TCS examination of L-CI was higher than that of the first (*P* = 0.023).

### Logistics regression analysis

The demographic differences of 172 patients, including age of onset, age of first treatment, and smoking history, were statistically significant between groups and were thus included in the logistics regression analysis. Although the difference in the change in the midbrain area within the group at the first and last examination of F-CI was statistically significant, there were differences in the application of the midbrain area in our cross-sectional study. Therefore, it is difficult to consider the midbrain area as an index to measure the disease progress of patients. SNH is a relatively qualitative imaging index. As a typical ultrasound manifestation of PD patients, it can represent the value of TCS in PD examination. Therefore, we only included the different ultrasound grading of SN in the logistics regression analysis.

#### Logistics regression analysis of the demographics and clinical symptoms of F-CI and N-CI

The results showed that the age of onset (*P* = 0.19), age of first treatment (*P* = 0.31), smoking history (*P* = 0.07), anxiety (*P* = 0.35), and depression (*P* = 0.51) were not independent risk factors for CI in PD patients at the first treatment.

**Fig. 4:**
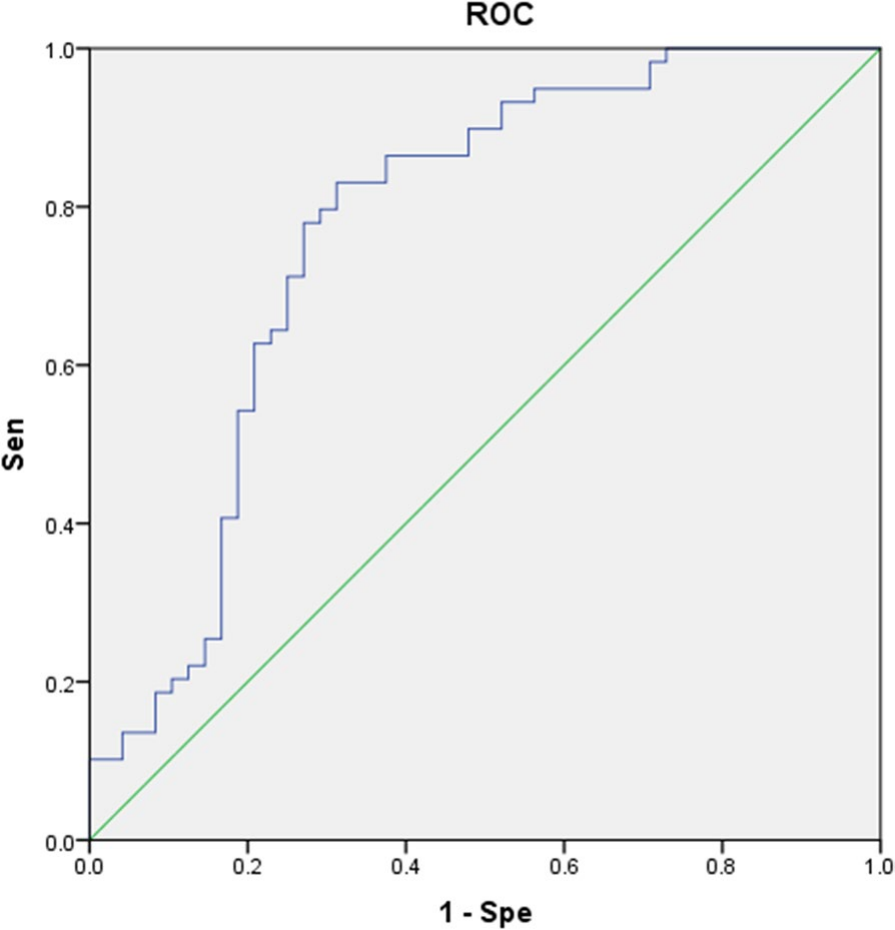
ROC obtained by clinical and demographic logistics regression analysis of F-CI and N-CI. AUC = 0.770 (95%CI: 0.675–0.865)

The ROC was established according to the logistics regression analysis results of F-CI and N-CI (Fig. 4), and the AUC was 0.770. The sensitivity and specificity of identifying PD patients with CI at the first treatment were 83.10 and 68.70%, respectively.

#### Logistics regression analysis of demographics, first clinical symptoms, and first ultrasound grading of L-CI and N-CI

There were statistically significant differences in depression (OR 5.58, 95% CI 1.63-19.07, *P* = 0.01), sexual dysfunction (OR 0.15, 95% CI 0.04-0.64, *P* = 0.01), and olfactory dysfunction (OR 0.26, 95% CI 0.09-0.70, *P* = 0.01) between L-CI and N-CI patients, indicating that these signs are independent risk factors in identifying PD patients without CI at the first treatment and whether CI will occur later.

**Fig. 5:**
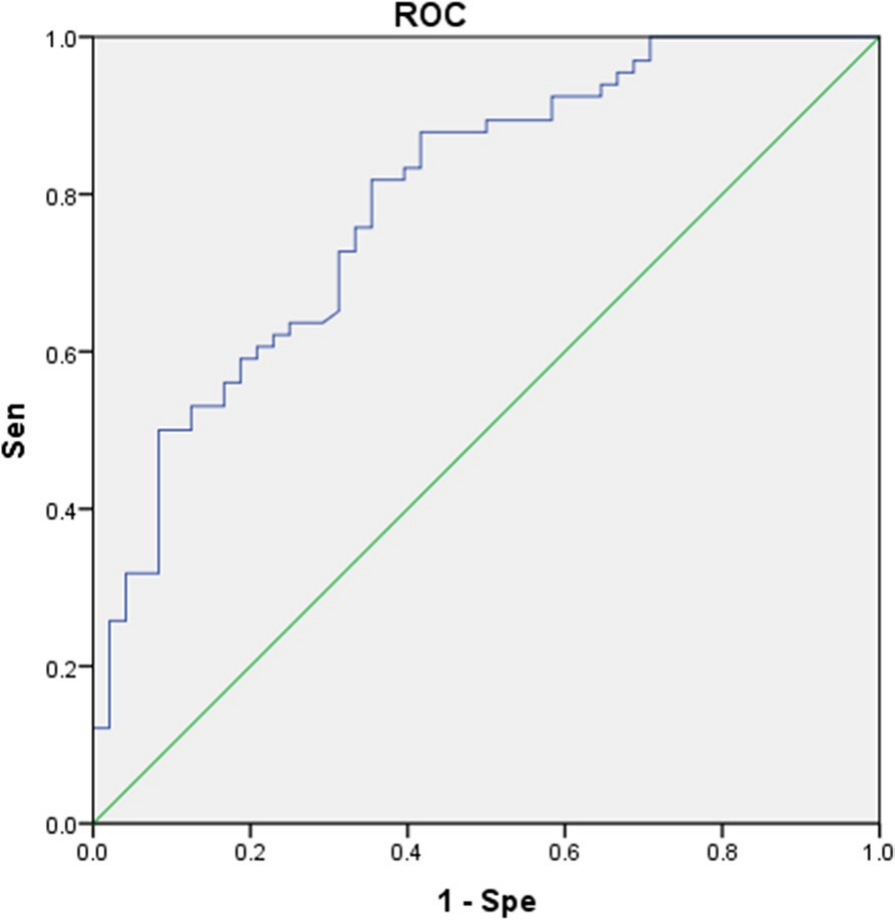
ROC obtained by clinical, demographic and ultrasonic grading logistics regression analysis of L-CI and N-CI at the first treatment. AUC = 0.793 (95% CI 0.710 - 0.875)

The ROC was established according to the logistics regression analysis results of L-CI and N-CI (Fig. 5), and the AUC was 0.793. The sensitivity and specificity of predicting CI in PD patients were 81.8 and 64.6%, respectively.

#### Logistics regression analysis of cumulative clinical symptoms, last ultrasound grading, and demographics of patients with L-CI and N-CI at the last treatment

Only memory deficit (OR 3.86, 95% CI 1.49-9.98, *P* = 0.01) was an independent risk factor for CI in PD patients, while the last ultrasound grading (OR 1.02, 95% CI 0.42-2.44, *P* = 0.97) was not an independent risk factor.

**Fig. 6:**
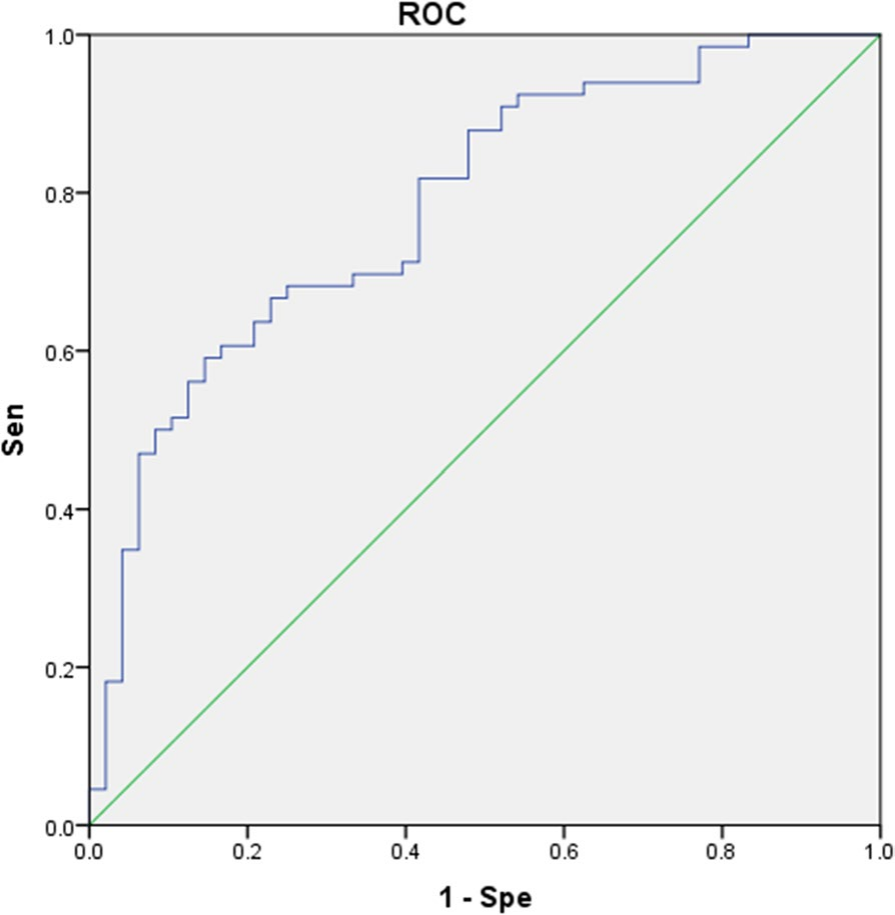
ROC obtained by clinical, demographic, and ultrasound grading logistics regression analysis of L-CI and N-CI at the last treatment. AUC = 0.783 (95%CI: 0.699–0.868)

The ROC was established according to the logistics regression analysis results of the L-CI and N-CI (Fig. 6), and the AUC was 0.783. The sensitivity and specificity of identifying PD patients with CI at the last treatment were 59.1 and 85.4%, respectively.

### Risk factors for PDD in patients with cognitive impairment

Although it has been reported that patients with CI will eventually meet the diagnostic criteria of dementia [10], only some patients were finally diagnosed with PDD or dementia during the follow-up period in our study. Therefore, we analysed the clinical and ultrasound odds ratios (ORs) of PDD and non-PDD to identify the risk factors for dementia in patients with CI. An OR = 1 indicates that this factor has no effect on the occurrence of disease; OR > 1 indicates that the factor is a risk factor; OR < 1 indicates that the factor is a protective factor.

In PD patients with CI, the risk of dementia increased 5.882 times with RCP. Moreover, rigidity and olfactory dysfunction are also risk factors for dementia, 2.091- and 1.987-times higher than in N-PDD patients, respectively. However, drinking history, somnipathy, and sexual dysfunction were protective factors for dementia in PD patients with CI (Table 5).

**Table 5:** Comparison of clinical data and ultrasonic grading between PDD and N-PDD patients

	PDD (*n* = 27)	N-PDD (*n* = 97)	OR
Gender (Male)	19	61	1.402
Smoking history	17	70	0.656
Drinking history	1	12	0.272
Hyperlipidemia	6	19	1.173
Diabetes	8	26	1.150
Hypertension	12	35	1.417
Bradykinesia	27	97	NA
Tremor	22	85	0.621
Rigidity	20	56	2.091
Memory deficit	22	76	1.216
RCP	15	17	5.882
Somnipathy	5	37	0.369
Anxious	20	71	1.046
Depressed	18	75	0.587
Nonchalance	3	8	1.390
Constipation	21	73	1.151
Hyperhidrosis	0	4	NA
Postural hypotension	11	38	1.067
Dysuria	16	65	0.716
Salivation	6	20	1.100
Sexual dysfunction	1	9	0.350
Olfactory dysfunction	16	41	1.987
Pain	6	29	0.670
Spasm	0	0	NA
Numbness	0	6	NA
RLS	2	5	1.472
SNH	16	53	1.208

## Discussion

In this study, it was clear that there are differences in the age of onset, age of first treatment, and smoking history between patients with CI and N-CI. The older the age of PD onset, the greater the likelihood of developing CI. When the age of first treatment in PD patients was ≥61 years, the sensitivity and specificity of diagnosing CI were 77.40 and 66.70%, respectively. At present, studies have confirmed that age is the most relevant predictor of CI in PD patients, and our results are consistent with this report [20, 21]. Although the grading of the last TCS examination of L-CI was higher than that of the first, the logistic regression analysis showed that the results of TCS were not an independent predictor of PD patients with CI. However, in addition to observing SNH, cerebral blood flow status can also be assessed by TCD, including the pulsatility index, resistivity index, and peak systolic blood flow velocity, which have been recently found to be useful for the early detection of both cognitive impairment and late-onset depression [22, 23]. When analysing the OR of dementia in PD patients, it was found that smoking history was a protective factor against dementia in patients with CI (OR < 1). Some studies have shown that there is a significant negative correlation between smoking and PD. It is believed that nicotine affects dopaminergic activity by acting on the nicotine receptor at the dopaminergic end and regulating the release of dopamine, but the role of other tobacco components cannot be excluded [24, 25].

In this study, the proportion of PD patients with CI accompanied by depression at the first treatment was 36.21% (21/58), which was similar to the results of a previous meta-analysis (25 ~ 40%) [26]. Although depression is a risk factor for CI (even dementia) in PD patients [27], patients with depression can also progress to CI without PD [28]. Therefore, it cannot be conclusively determined whether patients with PD who are suffering from depression and CI are experiencing PD with CI or depression with CI (i.e., false CI). In our study, there were significant differences between olfactory dysfunction at the first treatment of N-CI and L-CI, and the results of logistic regression analysis showed that olfactory dysfunction, depression, and sexual dysfunction were independent risk factors for CI in PD patients. Logistic regression analysis showed that the sensitivity and specificity of predicting cognitive impairment in PD patients were 81.80 and 64.60%, respectively. There are relatively few studies on sexual dysfunction in PD. At present, studies have reported that PD patients are more likely to suffer from sexual dysfunction than normal people [29, 30]. In our study, we found that sexual dysfunction is an independent predictor of N-CI in PD patients, and sexual dysfunction is a protective factor for dementia in patients with CI (OR = 0.350). Therefore, we can speculate that sexual dysfunction plays a protective role in the progression of PD, which may be related to the level of sex hormones [31, 32]. Cognitive impairment is the most common and important NMS. The timing, characteristics, and rate of CI vary greatly among individuals with PD. It is very important to actively prevent PD patients who may progress to CI, and lifestyle factors such as diet play an important role in preventing cognitive decline in later life. Recent research shows that phenolic acids in the diet may play a role in neuroprotective and cognitive activities. Additionally, acetyl L-carnitine (ALC) is also effective at slowing cognitive decline, and lifestyle factors such as diet play an important role in preventing CI in later life. Recent research shows that phenolic acids in the diet may play a neuroprotective and cognitive promoting role [33]. At the same time, acetyl L-carnitine (ALC) also has a good effect in slowing down cognitive decline [34]. Moreover, several studies have shown that CI is a precursor of PDD [35–37]. Therefore, it is important to identify which clinical and imaging manifestations may be related to the occurrence of dementia in PD patients with CI. We used ORs to identify the risk factors for dementia in patients with CI. OR > 1 indicates that the sign is a risk factor for dementia in patients with CI. OR < 1 indicated a protective factor. In our study, male sex, hyperlipidaemia, hypertension, diabetes, rigidity, memory deficit, RCP, anxiety, apathy, constipation, postural hypotension, salivation, olfactory dysfunction, RLS, and SNH were risk factors for PDD in patients with CI.

## Limitations

First, the duration of this precursor stage was different, resulting in some patients’ clinical symptoms deviating from the real situation in the process of history taking, and the possibility of interindividual variability between the two sonographers cannot be excluded. Some patients with a short follow-up period may not have CI or dementia at present, but this does not mean that these patients will not develop CI or dementia in the future. Second, many patients were not treated according to the doctor’s order during discharge, which makes us unable to accurately evaluate the therapeutic effect of drugs. Finally, this study is a retrospective analysis; therefore, selection bias cannot be avoided.

## Conclusions

It has good diagnostic efficiency through TCS with clinical and demographic characteristics to predict CI in PD. In our study, PD patients with CI were older, and most had a history of smoking. Impaired memory, depression, sexual dysfunction and olfactory dysfunction in NMS were independent risk factors for PD patients with CI, and RCP was the most important risk factor for PDD.

## Data Availability

The datasets generated during and/or analyzed during the current study are available from the corresponding author upon reasonable request.

## Abbreviations

PD: Parkinson’s disease
MS: Motor symptoms
NMS: Non-motor symptoms
TCS: Transcranial sonography
MCI: Mild cognitive impairment
SN: Substantia nigra
SNH: Substantia nigra hyper-echogenicity
N-CI: Non-cognitive impairment
F-CI: First-cognitive impairment
L-CI: Last-cognitive impairment
PDD: Parkinson’s disease dementia
RLS: Restless legs syndrome
RCP: Reduced computing power
ET: Essential tremor
PSP: Progressive supranuclear palsy
MSA: Multiple system atrophy
CNS: Central nervous system
OR: Odds ratio
ROC: Receiver operating characteristic curve
AUC: Area under the ROC curve
SD: Standard deviation
